# The Impact of Intervention Modality on Mortality Outcomes in Patients With Hemorrhagic Strokes: A Meta-Analysis

**DOI:** 10.7759/cureus.96820

**Published:** 2025-11-14

**Authors:** Sydney MacGregor, Varun Soti

**Affiliations:** 1 Emergency Medicine, Lake Erie College of Osteopathic Medicine, Elmira, USA; 2 Pharmacology and Therapeutics, Lake Erie College of Osteopathic Medicine, Elmira, USA

**Keywords:** emergency medicine, intracerebral hemorrhage, medical management, meta-analysis, mortality outcomes, surgical management

## Abstract

Introduction: Hemorrhagic strokes significantly contribute to stroke-related mortality. Despite advancements in critical care and neuroimaging, optimal management strategies for these strokes remain debated. Medical management focuses on stabilizing physiological parameters and preventing hematoma expansion, whereas surgical evacuation directly addresses mass effect and lowers intracranial pressure (ICP). This meta-analysis evaluates the impact of various intervention modalities and compares surgical versus medical interventions on mortality outcomes in patients with hemorrhagic strokes.

Materials and methods: Per the Preferred Reporting Items for Systematic Reviews and Meta-Analyses guidelines, a literature search was conducted on PubMed, BioMed Central, the *New England Journal of Medicine*, and the *American Heart Association* journals. Quality assessment was performed using the Newcastle-Ottawa scale. Randomized and nonrandomized controlled trials, case series and reports, and prospective, observational, and retrospective studies reporting mortality outcomes in patients treated with medical and/or surgical interventions were included. The primary outcome assessed was overall mortality. Odds ratios (ORs) with 95% confidence intervals (CIs) were pooled using a random-effects model. Statistical heterogeneity was evaluated using the Q test and I² statistic, while publication bias was assessed using Duval and Tweedie’s Trim and Fill method.

Results: Seventeen studies, encompassing 9077 patients, were included, with nine evaluating surgical management and eight examining medical management. Pooled analysis showed a modest but statistically significant reduction in mortality across all intervention groups compared with control (p = 0.05; OR: 1.218; 95% CI: 1.000-1.483). Subgroup analysis indicated that surgical interventions significantly decreased mortality compared with medical management (p = 0.009; OR: 1.273; 95% CI: 1.057-1.489), suggesting a survival benefit. In contrast, medical management did not achieve statistical significance (p = 0.606). Moderate heterogeneity was noted (I² = 51.997%), and sensitivity analysis confirmed the robustness of the findings.

Discussion: This study shows that surgical and medical management strategies enhance survival in patients with hemorrhagic stroke, with surgical interventions showing a notable mortality advantage, particularly in cases of large hematomas or rapid neurological decline. Advanced surgical methods such as decompressive craniectomy and thrombolysis-assisted evacuation help reduce ICP and secondary brain injury. While medical therapies aim to prevent hematoma expansion, their impact on mortality has been limited. These findings may encourage clinicians to adopt surgical interventions in high-risk patients to optimize outcomes. Moreover, medical management strategies need reevaluation due to their limited effect on mortality. Future research should focus on the timing of interventions and enhancing functional recovery through multidisciplinary rehabilitation.

Conclusion: This study highlights that both surgical and medical management are critical to improving outcomes in hemorrhagic strokes. Surgical interventions, guided by hematoma characteristics, provide a survival advantage in selected cases. However, individualized treatment planning remains essential, emphasizing the need for optimized medical care, including intensive blood pressure control and comprehensive supportive management.

## Introduction and background

Stroke is a major global health concern, causing substantial death, disability, and strain on healthcare systems. According to the World Health Organization, stroke is the second most common cause of mortality globally, with approximately 15 million new cases reported annually [1]. Understanding stroke is critical to healthcare, as early recognition and intervention can significantly improve patient outcomes. Stroke is classified into two primary types: ischemic and hemorrhagic. Ischemic strokes, which account for approximately 80% of all stroke cases, occur when there is an obstruction of blood flow to the brain. This obstruction can result from thrombosis (a clot that forms within a blood vessel), embolism (a clot that travels from another part of the body), or systemic hypoperfusion (a general reduction in blood flow due to conditions such as heart failure) [2]. In contrast, hemorrhagic strokes, although less common (accounting for 10% to 20% of all strokes), are caused by bleeding in the brain and can lead to greater mortality and disability because of the complexity and severity of the underlying pathology [3].

Despite improvements in medical care and preventive strategies, the global burden of stroke continues to rise. Between 1990 and 2010, the absolute incidence of global strokes increased by 47%, with a significant rise in hemorrhagic stroke cases [4]. The lifetime risk of stroke for adults over 25 years of age is approximately 25% [5]. Stroke incidence and mortality vary globally. Low- and middle-income countries face higher burdens due to limited healthcare access, poor hypertension management, and genetic predisposition [6]. In China, for instance, the age-standardized prevalence of stroke is estimated at 1,115 per 100,000 person-years, with an annual incidence rate of 247 per 100,000 person-years and a mortality rate of 115 per 100,000 person-years [7].

In the United States, approximately 795,000 strokes occur annually, with 610,000 being first-ever strokes and 185,000 being recurrent events [8]. The southeastern United States, referred to as the “stroke belt,” exhibits higher incidence and mortality rates compared with other regions, primarily due to a higher prevalence of hypertension, diabetes, and obesity [9]. Among racial and ethnic groups, Black and Hispanic Americans experience higher rates of stroke compared with White Americans, attributed to increased rates of intracranial atherosclerosis and small vessel strokes [10].

Ischemic strokes result from the blockage of cerebral arteries, leading to tissue infarction due to reduced oxygen and nutrient supply [11]. These strokes may occur because of thrombosis (for example, large vessel atherosclerosis), embolism (such as cardiogenic emboli), or systemic hypoperfusion (for example, cardiac arrest) [12]. Conversely, hemorrhagic strokes result from the rupture of blood vessels in the brain, leading to bleeding into the brain parenchyma or surrounding cerebrospinal fluid (CSF) spaces. They can be categorized into several subtypes: intracerebral hemorrhage, subarachnoid hemorrhage (SAH), subdural hemorrhage (SDH), and epidural hemorrhage (EDH) [13]. Intracerebral hemorrhage involves bleeding directly into the brain parenchyma, most often resulting from hypertensive damage to small arteries [14]. SAH occurs when bleeding enters the subarachnoid space, typically resulting from aneurysmal rupture or vascular malformations. SDH and EDH generally occur secondary to trauma, resulting in venous or arterial bleeding between meningeal layers [15].

Among hemorrhagic strokes, intracerebral hemorrhage and SAH are the predominant spontaneous subtypes. Intracerebral hemorrhage accounts for approximately 10% to 15% of all strokes in high-income countries and up to 29% in low- and middle-income countries [16]. Common causes include hypertension, cerebral amyloid angiopathy (the accumulation of amyloid protein in the cerebral blood vessel walls), arteriovenous malformations, bleeding disorders, and illicit drug use (especially amphetamines and cocaine) [17,18]. In contrast, SAH is most frequently caused by the rupture of intracranial aneurysms or arteriovenous malformations. SDH and EDH are caused by traumatic injury; however, they remain clinically significant due to their acute neurological consequences [15].

The clinical presentation of hemorrhagic strokes varies depending on the subtype and the location of the bleeding. In general, symptoms may include sudden-onset headache, vomiting, focal neurological deficits, altered level of consciousness, and seizures. Intracerebral hemorrhage often manifests with focal neurological deficits such as hemiparesis or aphasia, depending on the site of bleeding [19,20]. SAH typically presents with a sudden, severe “thunderclap” headache accompanied by neck stiffness, photophobia, or loss of consciousness. SDH and EDH frequently present with signs of increased intracranial pressure or focal deficits following trauma [15].

Prompt neuroimaging is required to confirm the diagnosis of hemorrhagic stroke. Noncontrast computed tomography (CT) remains the gold standard for identifying and differentiating hemorrhagic lesions, allowing for rapid delineation of hematoma location, size, and mass effect [21]. Magnetic resonance imaging may be utilized in select cases to further evaluate underlying structural causes. For suspected SAH, CT angiography or digital subtraction angiography helps identify ruptured aneurysms or arteriovenous malformations [22].

The severity of hemorrhagic strokes is assessed using specific scoring systems. The intracerebral hemorrhage score incorporates the Glasgow Coma Scale, hematoma volume, age, and infratentorial location. In contrast, the Hunt-Hess and World Federation of Neurological Surgeons (WFNS) scales are utilized for SAH to grade clinical severity and predict outcomes. These scales guide prognosis and treatment planning [23-25].

Management strategies for hemorrhagic strokes focus on stabilizing the patient, controlling bleeding, reducing intracranial pressure (ICP), and preventing secondary injury. In cases of intracerebral hemorrhage, aggressive blood pressure control, with a target of 140-160 millimeters of mercury, is recommended using agents such as nicardipine, labetalol, or clevidipine [26]. For patients on anticoagulant therapy, reversal agents including prothrombin complex concentrate, vitamin K, or direct oral anticoagulant antidotes (andexanet alfa, idarucizumab) are employed [27,28]. In instances of elevated ICP, hyperosmolar therapy (mannitol or hypertonic saline), sedation, and CSF drainage can help reduce pressure [29]. In SAH, the mainstay of therapy includes early aneurysm repair via surgical clipping or endovascular coiling and the administration of nimodipine to prevent vasospasm [30].

Surgical interventions are often indicated in hemorrhagic strokes where there is significant mass effect or hydrocephalus. In cases of intracerebral hemorrhage, options include craniotomy, stereotactic aspiration, or minimally invasive evacuation. Decompressive craniectomy may be performed for large hematomas or refractory ICP elevation. For SAH, surgical clipping or endovascular coiling of ruptured aneurysms remains standard care, while ventriculostomy can relieve hydrocephalus in both intracerebral hemorrhage and SAH [30].

Despite advances in management, hemorrhagic strokes continue to exhibit high mortality and morbidity rates. The mortality rate for intracerebral hemorrhage remains approximately 30% to 40% within the first month, with only about 20% of survivors achieving functional independence at six months [31]. SAH carries a similar burden, with up to one-third of patients dying within a month and many survivors experiencing cognitive and neurological deficits [32]. Prognosis largely depends on hematoma size, bleeding location, initial neurological status, and the rapidity of intervention [33].

Given the persistent mortality associated with hemorrhagic strokes, the optimal management strategy remains the subject of ongoing research. Although surgical interventions may benefit select cases, the relative advantages over medical therapy are still debated across different subtypes. This meta-analysis evaluates the efficacy of surgical versus medical management through studies encompassing all forms of hemorrhagic stroke, focusing on mortality outcomes to inform evidence-based clinical practice and improve patient care.

## Review

Methods 

We adhered to the Preferred Reporting Items for Systematic Reviews and Meta-Analyses (PRISMA) guidelines and employed the Newcastle Ottawa Scale for quality assessment [34-35].

Search Strategy

We searched literature using digital databases, including PubMed, BioMed Central, the New England Journal of Medicine, and American Heart Association Journals. Our key search terms included “hemorrhagic strokes,” “surgical management,” “medical management,” and “mortality.” In particular, we focused on various types of “hemorrhagic strokes,” examining their “surgical” and “medical treatments” alongside related “mortality.” The specific terms we used included combinations of “intracerebral hemorrhage,” “subarachnoid hemorrhage,” “subdural hemorrhage,” and “epidural hemorrhage,” along with their respective “surgical” and “medical treatments” and associated “mortality.” To enhance our analysis, we reviewed the reference lists of the selected articles for additional studies. We ensured a comprehensive analysis by not restricting our search based on country or publication date, and we assigned a level of clinical evidence to each selected study [36].

Study Selection

The inclusion criteria included the following: (i) randomized controlled trials (RCTs), nonrandomized controlled trials case reports, case studies, prospective controlled studies (PCS), observational studies, and retrospective studies (RS), that reported mortality rates in patients aged between 16 and 86 with hemorrhagic stroke who received surgical and/or medical intervention; (ii) studies published in English. The exclusion criteria included the following: (i) commentaries; (ii) systematic reviews and meta-analyses.

Data Collection

Two reviewers conducted independent searches using the terms outlined earlier. They then collaborated to eliminate duplicate studies, identifying those that met the inclusion criteria for final selection. They compiled general details about each included study, including the publication year, study design, sample size, patient age and gender, and treatment modalities. The primary outcome focused on from each study was the mortality rate. Additionally, they gathered information on the various treatment groups, which included surgical interventions, drug therapies, and minimally invasive or bedside procedures, to facilitate subgroup analysis.

Statistical Analysis

We conducted the data analysis using Comprehensive Meta-Analysis, version 4.0.00 (Biostat, Inc., Englewood Cliffs, New Jersey). To assess mortality following treatment, we calculated odds ratios (OR) with a 95% confidence interval (CI), considering a probability (p) value of 0.05 or less as statistically significant. We performed a subgroup analysis to evaluate whether treatment modality (surgical versus medical management) affected the outcomes we investigated. The degree of statistical heterogeneity was quantified using the I² statistic, and the presence of heterogeneity was confirmed through the Q-test. Our analysis yielded a Q-value of 33.331 with a p-value of 0.007, resulting in an I² value of 51.997%. Due to the significant Q-value and I² exceeding 50%, we employed a random-effects model for subsequent analyses. To assess publication bias, we utilized Duval and Tweedie’s Trim and Fill method [37].

**Figure 1:**
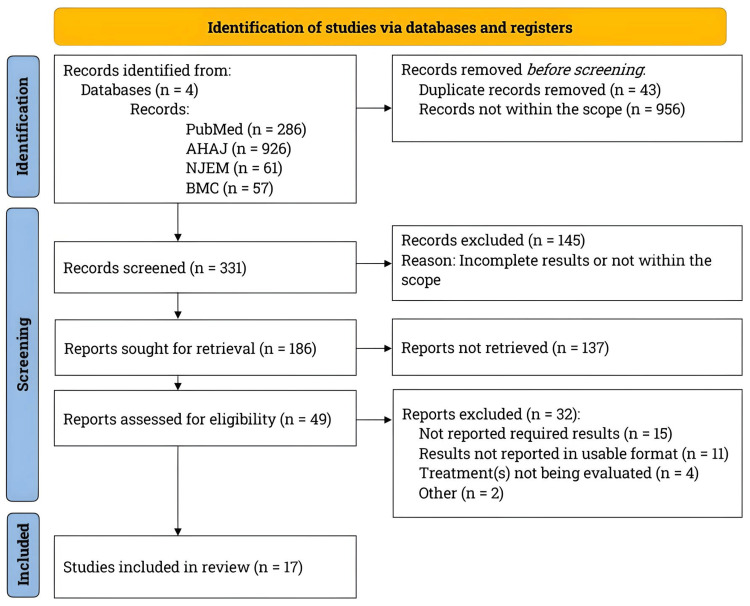
Overview of the literature search and study selection process. The PRISMA flowchart depicts the literature search and study selection process. Following PRISMA guidelines, we searched for studies involving patients with intracerebral hemorrhage that compared surgical interventions to medical treatments, with a primary focus on mortality as the primary outcome measure. AHAJ: American Heart Association Journals; BMC: BioMed Central; n: Number; NEJM: New England Journal of Medicine; PRISMA: Preferred Reporting Items for Systematic Reviews and Meta-Analyses. Fotor (Chengdu Everimaging Science and Technology Co., Ltd., Chengdu, China), an online artificial intelligence application, was utilized to enhance the image’s resolution.

The PRISMA flowchart depicting the literature search and study selection process is shown in Figure 1.

We employed a systematic approach to select relevant literature and methodologically gather and analyze data on the management and mortality rates associated with hemorrhagic strokes. Our process involved thorough searches in multiple digital databases and carefully considering inclusion and exclusion criteria for studies. We ensured that data was collected accurately, focusing on key details and outcomes, allowing us to analyze the effectiveness of various treatment modalities. The following section presents the results derived from our comprehensive literature search and data analysis.

Results

The results of our meta-analysis are as follows: A comprehensive literature search identified 17 studies that fulfilled the inclusion criteria. Among these studies, 14 were RCTs, two were RS, and one was a PCS. The total number of patients included in these studies was 9,077. While some studies had dual treatment arms addressing medical and surgical management, they were primarily categorized into two overarching groups based on the predominant treatment approach: eight focused on “medical management” and nine on “surgical management.”

Table 1 summarizes the general characteristics of these studies, including patient demographics and treatment modalities.

**Table 1:** General characteristics of the studies included in this meta-analysis. BP: blood pressure, CSF: cerebrospinal fluid, CT: conservative treatment, DC: decompressive craniectomy, EVD: extra-ventricular drain, MIR: minimally invasive removal, MISPT: minimally invasive stereotactic puncture therapy, MISTIE: minimally invasive surgery with thrombolysis in intracerebral haemorrhage evacuation, PCS: prospective controlled study, RCT: randomised controlled trial, RS: retrospective study, rt-PA: recombinant tissue plasminogen activator.

Authors	Publication Year	Study Design	Groups	Treatment Modality	Sample Size	Average Age (Years)	% Female
Miah et al. [38]	2023	RCT	Dexamethasone vs. Surgery (standard care)	Medical	252	75	23
Hutchinson et al. [39]	2020	RCT	Dexamethasone vs. Placebo	Medical	748	74	26
Walker et al. [40]	2020	RS	Tranexamic acid vs. Standard care	Medical	71	25	0
Sprigg et al. [41]	2018	RCT	Tranexamic acid vs. Placebo	Medical	2,325	69	44
Haley et al. [42]	1997	RCT	Tirilazad mesylate vs. Placebo	Medical	599	51	68
Qureshi et al. [43]	2016	RCT	Intensive BP lowering vs. Standard care	Medical	1,000	62	38
Mendelow et al. [44]	2005	RCT	Early surgery vs. Initial CT	Surgical	1,033	62	43
Hutchinson et al. [45]	2023	RCT	DC vs. Craniotomy (standard care)	Surgical	450	49	21
Zhou et al. [46]	2011	PCS	MISPT vs. craniotomy	Surgical	168	58	35
Mendelow et al. [47]	2013	RCT	Early Surgery vs. Initial CT	Surgical	601	64	43
Wolf et al. [48]	2023	RCT	Lumbar CSF drainage vs. Standard care	Medical	287	55	69
Mayer et al. [49]	2005	RCT	Recombinant activated factor VII vs. Placebo	Medical	199	66	40
Phillips et al. [50]	2011	RS	Early surgery vs. Initial CT	Surgical	459	53	65
Litrico et al. [51]	2013	RCT	EVD with rt-PA vs. EVD (standard care)	Surgical	19	56	37
Hanley et al. [52]	2019	RCT	MISTIE vs. Standard care	Surgical	506	62	38
Wu et al. [53]	2023	RCT	Stellate ganglion block vs. Standard care	Medical	60	52	55
Pradilla et al. [54]	2024	RCT	Early MIR vs. Standard care	Surgical	300	63	50

The Newcastle-Ottawa Scale was employed to assess the quality of the included studies. Notably, 14 of the 17 studies received scores of 6 or 7 (out of 7), indicating that the majority were of high quality. Table 2 provides a detailed quality assessment of the studies according to the Newcastle-Ottawa Scale, highlighting aspects such as selection, comparability, and outcome assessment. 

**Table 2:** Quality assessment of the included studies per the Newcastle-Ottawa scale. *Represents a score of 1. – Represents a score of 0.

Authors (Years)	Representativeness of Exposed Cohort	Ascertainment of Exposure	Outcome of Interest (Not Present at Start)	Comparability by Study Design	Assessment of Outcome	Follow-up Length	Adequacy of Follow-up	Score
Miah et al. (2023) [38]	*	*	*	*	*	–	*	6
Hutchinson et al. (2020) [39]	*	*	*	*	*	*	*	7
Walker et al. (2020) [40]	–	*	*	*	*	*	*	6
Sprigg et al. (2018) [41]	*	*	*	*	*	–	*	6
Haley et al. (1997) [42]	*	*	*	*	*	–	*	6
Qureshi et al. (2016) [43]	*	*	*	*	*	*	–	6
Mendelow et al. (2005) [44]	*	*	*	*	*	*	*	7
Hutchinson et al. (2023) [45]	*	*	*	*	*	*	*	7
Zhou et al. (2011) [46]	*	*	*	*	*	*	–	6
Mendelow et al. (2013) [47]	*	*	*	*	*	*	*	7
Wolf et al. (2023) [48]	*	*	*	*	*	*	*	7
Mayer et al. (2005) [49]	*	*	*	*	*	–	–	5
Phillips et al. (2011) [50]	–	*	*	*	*	–	*	5
Litrico et al. (2013) [51]	*	*	*	*	*	–	*	6
Hanley et al. (2019) [52]	*	*	*	*	*	*	*	7
Wu et al. (2023) [53]	–	*	*	*	*	–	*	5
Pradilla et al. (2024) [54]	*	*	*	*	*	*	*	7

Overall Mortality Analysis

**Figure 2:**
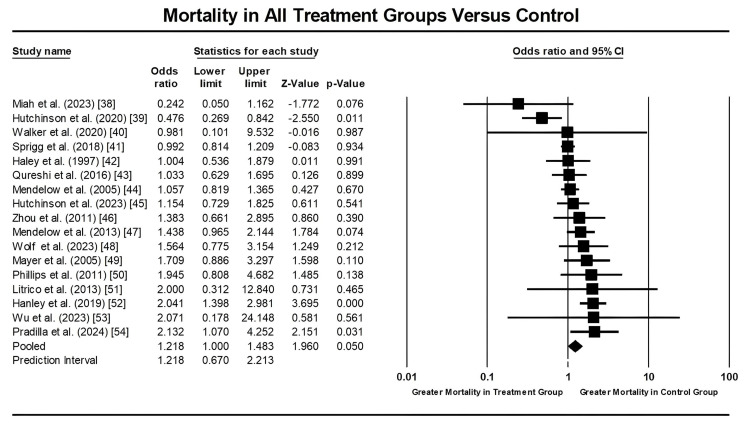
Forest plot showing mortality rates in patients with hemorrhagic strokes undergoing treatment (medical and/or surgical) compared to control (standard care or placebo). Data from references [38-54].

A comprehensive analysis of 17 studies, encompassing 9,077 patients, examined all-cause mortality among individuals hospitalized for hemorrhagic strokes. The findings revealed a borderline significant difference in mortality rates between treatment and control groups, with a p-value of 0.05 (CI: 1.000-1.483). Specifically, patients who received surgical or medical treatment (treatment group) exhibited a lower mortality frequency than those who received either a placebo or standard care (control group), as illustrated in Figure 2.

**Figure 3:**
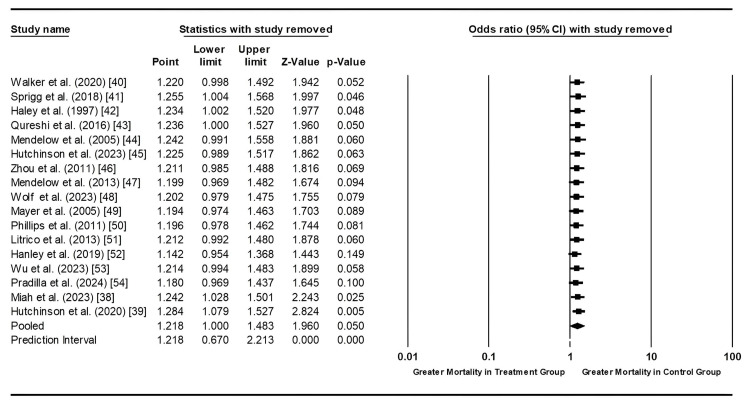
Sensitivity analysis of the studies showing mortality rates in patients with hemorrhagic strokes undergoing treatment (medical and/or surgical) compared to control (standard care or placebo). Data from references [38-54].

There was moderate heterogeneity among studies, as indicated by an I² value of 51.997%, necessitating a random-effects model. Therefore, we conducted a sensitivity analysis to ensure that no single study unduly influenced these results. The mean effect size was 1.218, with a p-value of 0.05, reinforcing these findings’ validity (see Figure 3).

Subgroup Comparison Between Surgical Versus Medical Management

**Figure 4:**
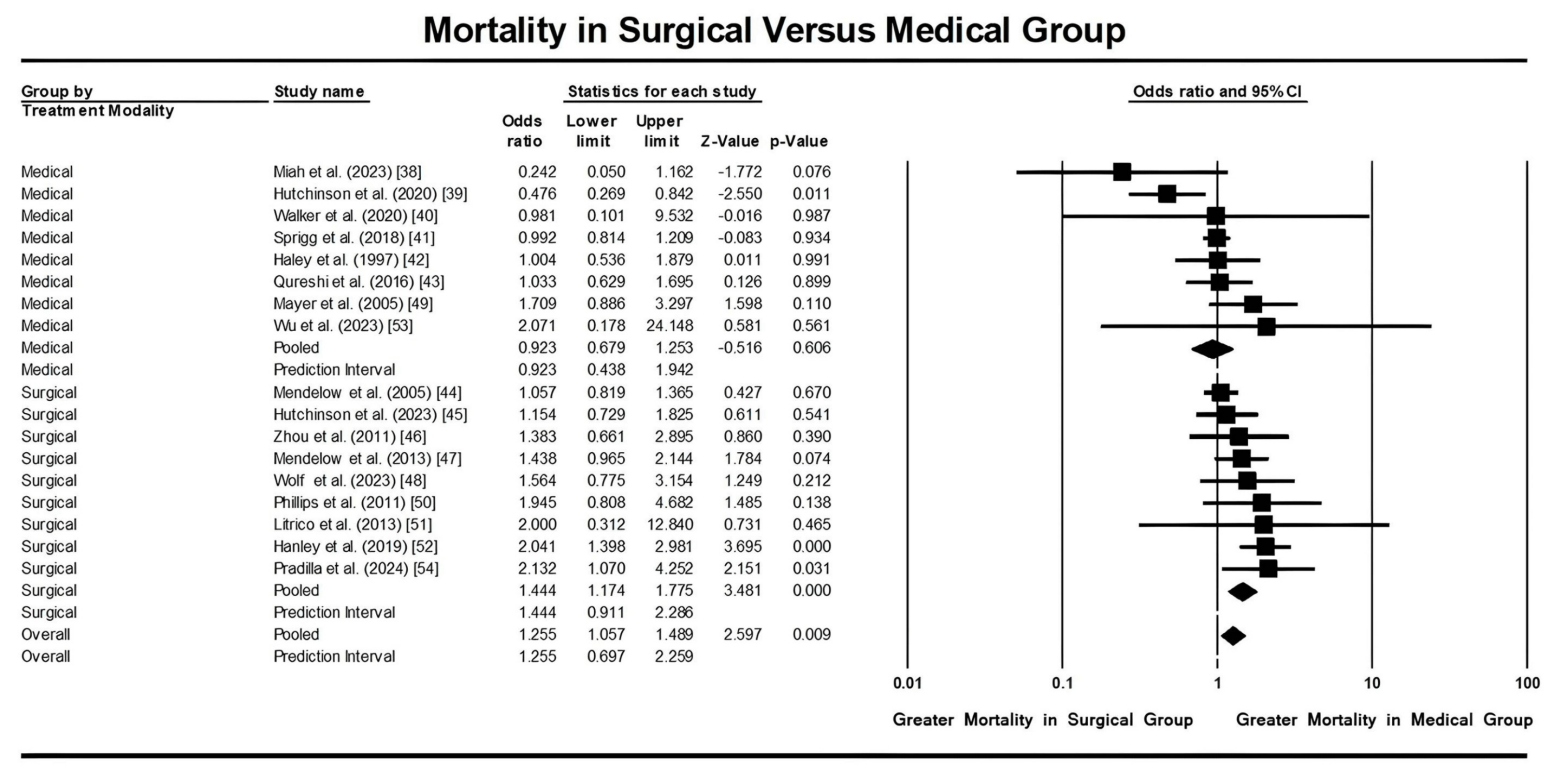
Forest plot showing mortality rates in patients with hemorrhagic strokes undergoing surgical intervention compared to medical management. Data from references [38-54].

We conducted a subgroup analysis comparing patients who received surgical management to those who underwent medical management. Surgical management encompassed treatments that required surgical intervention, while medical management included therapies that involved medication, bedside procedures, or innovative management strategies such as early blood pressure reduction therapy. The findings revealed that patients subjected to surgical management had a substantially lower mortality risk than those receiving medical management, with a statistically significant difference (p = 0.009, CI: 1.057-1.489) for the surgical group compared to the medical management group (p = 0.606, CI: 0.679-1.253), as demonstrated in Figure 4.

Publication Bias Assessment

**Figure 5:**
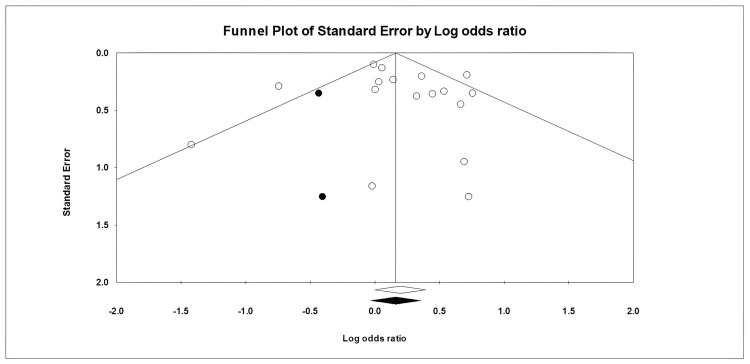
Funnel plot showing publication bias assessment.

We utilized Duval and Tweedie’s Trim and Fill method to assess the publication bias (see Figure 5).

In this analysis, open circles represent the included studies, while filled circles represent estimated studies that might not have been published. The studies with large effect sizes, meaning they showed stronger results, are located at the top of the plot, while those with smaller effect sizes are lower down the plot. Two studies were adjusted during this process using a random-effects model. The point estimate for the observed studies is 1.15 (CI: 1.03-1.29), and for the adjusted studies, it is 1.14 (CI: 1.02-1.27). Because these values are very close, they suggest that publication bias will not likely significantly impact this analysis.

Discussion and limitations 

The findings from this meta-analysis underscore the critical impact of treatment modality on mortality outcomes in patients with hemorrhagic strokes. Our pooled analysis of 17 studies, comprising RCTs, RS, and PCS, reveals that both medical and surgical interventions significantly reduce mortality compared with standard care or placebo. Surgical management demonstrates a statistically significant survival benefit (p = 0.009; OR: 1.273, 95% CI: 1.057-1.489) compared with medical management alone (p = 0.606). This aligns with historical data suggesting that, in selected patients, surgical evacuation of hematomas may alleviate mass effects, reduce ICP, and prevent secondary neuronal injury. Patients with superficial lobar hematomas or a deteriorating neurological status may benefit from early surgical intervention [44,47,52]. The success of these surgical interventions should reassure clinicians about their effectiveness in managing hemorrhagic strokes.

A systematic review of the studies we assessed for the subgroup analysis indicates that surgical modalities such as decompressive craniectomy, minimally invasive stereotactic puncture therapy, and minimally invasive surgery with thrombolysis (MISTIE) hold significant promise in reducing mortality compared with conservative medical management. The reviewed literature suggests that these surgical approaches decrease the immediate mortality risk and improve overall patient outcomes by addressing the underlying pathophysiology associated with hematomas [38-54]. This emphasis on the potential of surgical techniques should instill a sense of hope in patients regarding the future of hemorrhagic treatment.

Specifically, decompressive craniectomy involves the removal of a portion of the skull to allow for brain swelling, significantly alleviating pressure and improving cerebral perfusion. Minimally invasive procedures like stereotactic puncture therapy provide a less traumatic option for evacuating hematomas, reducing complications associated with larger surgical openings. On the other hand, MISTIE combines thrombolytic treatment with minimally invasive surgical techniques to effectively reduce clot volume without extensive surgical intervention, which may lower the incidence of herniation syndromes [38-54].

These surgical modalities are theorized to mitigate the pathophysiological sequelae of hematoma expansion, including herniation syndromes and compromised cerebral perfusion pressure. Early intervention using these techniques has been shown to stabilize the structural integrity of the brain, minimize secondary injury, and enhance patients’ recovery potential. However, it is essential to note that surgical interventions also carry potential risks and complications, such as infection, bleeding, and neurological deficits. Therefore, while conservative medical management may be appropriate in selected cases, the evidence strongly supports the preference for these surgical interventions in managing life-threatening intracranial hemorrhages [38-54].

In several trials, medical treatment arms incorporated novel interventions such as tranexamic acid, dexamethasone, recombinant factor VIIa, and established strategies like intensive blood pressure control. These agents were hypothesized to mitigate complications related to intracranial hemorrhages; however, their effectiveness in significantly reducing mortality lacks consistent statistical backing across studies. Tranexamic acid operates as an antifibrinolytic agent that lowers hematoma expansion by inhibiting the breakdown of fibrin in clots. Some evidence suggests it effectively curtails early hematoma growth, but this has not reliably translated into improved long-term survival outcomes [41]. Dexamethasone, a corticosteroid intended to reduce cerebral edema, may improve neurological outcomes; however, similar to tranexamic acid, its benefits regarding mortality remain ambiguous [38,39]. Additionally, recombinant factor VIIa, which promotes clotting, has been shown to reduce bleeding complications but has not uniformly demonstrated a mortality benefit across randomized trials [49].

Moreover, alternative medical interventions might be perceived as beneficial but lack evidence demonstrating their efficacy in significantly improving survival rates. These alternative approaches include osmotic diuresis, hypertonic saline administration, and hyperventilation strategies. Stringent blood pressure control is crucial in managing patients with hemorrhagic strokes. Osmotic diuretics, such as mannitol, effectively decrease ICP by drawing fluid out of the brain; however, their impact on mortality is not well defined. Similarly, hypertonic saline can rapidly increase serum osmolality, enhancing fluid redistribution through osmotic gradients, yet it also lacks robust evidence for a mortality benefit [43]. Controlled hyperventilation, aimed at reducing ICP through respiratory alkalosis, may offer temporary relief in specific acute scenarios but carries risks if not carefully monitored. Ultimately, while these interventions play vital roles in the acute management of hemorrhages, direct comparisons with surgical management emphasize the necessity of surgical interventions to reduce life-threatening outcomes substantially.

The MISTIE III trial, a significant study in the field, exemplifies the complexities of hybrid approaches in treating intracranial hemorrhages. This trial, which incorporates a hybrid model that combines catheter-based evacuation of hematomas with pharmacologic thrombolysis, did not demonstrate a significant mortality benefit [52]. This finding raises questions about patient selection and the timing of interventions. Previous studies have indicated that early intervention with surgical techniques can improve outcomes, suggesting delays in starting thrombolytic treatment may have impacted the trial’s effectiveness. Furthermore, this highlights the need for stringent selection criteria to identify candidates who would most benefit from such hybrid interventions. It emphasizes that nuances in study design and patient characteristics can significantly influence clinical decision-making in practice. These implications underscore the need for further research to refine patient selection criteria and optimize the timing of interventions in hybrid approaches.

Our results indicate a moderate but statistically significant heterogeneity in treatment outcomes, as demonstrated by the I² statistic of 51.997%. This variability stems from differences in patient demographics (age, gender, race, and locality, whether urban, suburban, or rural), hematoma characteristics, intervention timing, techniques, and co-management strategies, including the use of reversal agents or critical care support. For instance, patients with infratentorial hemorrhages or those experiencing rapid neurological decline may benefit more from prompt surgical decompression. However, these parameters were not consistently addressed across all included trials. Additionally, a lack of comprehensive reporting on various confounders, such as pre-existing conditions, history of prior trauma, surgical history, and neurological status upon admission and during multiple post-surgery intervals, limits our ability to perform additional subgroup statistical and regression analyses. Understanding these factors and their potential impact on treatment outcomes is crucial for making informed treatment decisions.

Despite the findings regarding mortality, it is crucial to recognize that this metric alone may not adequately reflect the clinical utility of an intervention. Functional outcomes, particularly those assessed through the modified Rankin Scale (mRS), are arguably more significant in patient-centered care. However, the inconsistency in reporting these data across the studies restricts our ability to evaluate the impact of interventions on functional independence. For instance, among the studies analyzed, eight utilized the mRS, a standardized tool for gauging the degree of disability or dependence in daily activities following a stroke. These studies included Miah et al. [38], Hutchinson et al. [39], Sprigg et al. [41], Mendelow et al. [44], Zhou et al. [46], Mendelow et al. [47], Hanley et al. [52], and Pradilla et al. [54], all of which assessed functional outcomes at various time points, typically 90 days, six months, or one year post-event. Their focus was on evaluating recovery and functional independence following interventions such as surgery, pharmacologic therapy, or minimally invasive procedures for hemorrhagic stroke.

Conversely, nine studies employed alternative instruments for assessing neurological or functional outcomes. Walker et al. [40], Haley et al. [42], Mayer et al. [49], Phillips et al. [50], Wolf et al. [48], and Wu et al. [53] utilized the Glasgow Outcome Scale (GOS) or its Extended version (GOS-E), which classifies patient recovery ranging from death, representing no recovery, to good recovery, based on overall neurological function. Additionally, Haley et al. [42] and Mayer et al. [50] incorporated the Barthel Index to evaluate daily living activities. Qureshi et al. [43] assessed quality of life and neurological deficits using the European Quality of Life 5-Dimension Scale and the National Institutes of Health Stroke Scale (NIHSS). Mendelow et al. [44] applied the NIHSS alongside the mRS for early neurological assessments, while Hutchinson et al. [45] used the GOS-E as the primary outcome measure following decompressive craniectomy. Moreover, Wolf et al. [48] and Wu et al. [53] reported functional status using modified or extended GOS variants to capture recovery following SAH hemorrhage. The variety of instruments used to measure functional outcomes highlights the need for standardized reporting to enhance our understanding of recovery trajectories and the efficacy of different interventions in clinical practice.

In addition, the timing of the intervention is a pivotal variable. Several trials emphasized the importance of early surgical evacuation, particularly within the first 12 hours of symptom onset, yet others allowed for considerable delays, potentially diluting the treatment effect. Similarly, medical interventions such as blood pressure reduction may exert differential effects based on timing and intensity. Another consideration pertains to the role of treatment withdrawal and goals-of-care discussions in influencing mortality statistics. Early withdrawal of life-sustaining treatments can artificially inflate mortality rates in observational studies, particularly when influenced by poor initial prognostication.

The influence of such practices remains unclear in the current meta-analysis but may represent an unmeasured confounder. While we sought to include only high-quality studies per the Newcastle-Ottawa Scale, variations in study design, sample sizes, and endpoint definitions remain limitations. Despite these challenges, several methodological strengths bolster the validity of our findings. The application of a random-effects model appropriately accounts for between-study variability. Furthermore, our sensitivity analysis confirms the robustness of the results, and the publication bias assessment using Duval and Tweedie’s Trim and Fill method indicates minimal bias, enhancing confidence in the observed effect sizes.

Clinical implications and future directions 

The outcomes of this meta-analysis are of significant importance in stroke care. They reveal that surgical interventions offer a measurable mortality benefit over medical management in patients with hemorrhagic strokes. This underscores the need for early surgical evaluation in clinically appropriate cases. Specifically, for patients with large hematoma volumes, rapid neurological deterioration, or superficial lobar bleeds, decompression could be a crucial step in preserving viable tissue and preventing secondary injury. The findings also stress the importance of individualized decision-making, considering patient age, comorbidities, hematoma location, and presentation timing to determine the most suitable management approach.

From a medical management perspective, the results underscore the urgent need to refine evidence-based protocols for hemorrhagic stroke care. Early and sustained blood pressure control remains a cornerstone of treatment, supported by prior large-scale randomized controlled trials. The prompt reversal of anticoagulation in eligible patients, particularly those on warfarin or direct oral anticoagulants, is equally critical to limit hematoma expansion. The emergence of adjunctive therapies, such as neuroprotective agents, antifibrinolytics like tranexamic acid, and anti-inflammatory interventions, further highlights the need for robust clinical trials to define their efficacy, safety, and timing in both acute and subacute phases of hemorrhagic stroke.

Beyond acute care, these findings underscore the necessity of addressing long-term recovery and prognostic considerations. Mortality and short-term functional independence are key endpoints; however, the broader continuum of post-stroke care extends to cognitive, emotional, and physical rehabilitation. Incorporating early multidisciplinary rehabilitation, including physiotherapy, occupational therapy, and cognitive retraining, has been shown to enhance neuroplasticity, improve mobility, and reduce long-term disability. Additionally, post-stroke cognitive decline and depression remain underrecognized predictors of poor quality of life and recurrent morbidity. Integrating standardized cognitive and psychological assessments into follow-up protocols can facilitate early intervention and improve reintegration outcomes.

These findings also carry implications for future research and clinical guideline development. Current evidence-based recommendations vary across international societies, partly due to heterogeneity in study designs, definitions of outcomes, and patient selection criteria. Therefore, future randomized controlled trials should aim to harmonize inclusion criteria, adopt standardized outcome measures such as the mRS, and stratify participants according to hematoma characteristics, location, and volume. There is also a critical need for longitudinal studies examining the durability of surgical and medical benefits, including effects on long-term neurological function, cognitive trajectory, and recurrent hemorrhage risk. Furthermore, integrating cost-effectiveness analyses, health equity assessments, and real-world implementation studies will enhance the translational value of future evidence.

Interdisciplinary collaboration among neurosurgeons, neurologists, intensivists, rehabilitation specialists, and health economists is beneficial and essential in developing comprehensive care pathways for stroke patients. The development and clinical integration of hemorrhagic stroke-specific decision-support algorithms and predictive modeling can further personalize treatment planning and guide discussions around prognosis, quality of life, and care goals. When implemented collectively, these strategies can bridge current evidence gaps, inform guideline updates, and optimize long-term outcomes for patients with hemorrhagic strokes.

## Conclusions

This meta-analysis underscores the critical roles of both surgical interventions and medical management in enhancing survival outcomes for patients suffering from hemorrhagic strokes. While surgical options may reduce mortality rates in select cases, treatment decisions must be individualized, taking into account factors such as hematoma size and location, the patient’s neurological status, comorbidities, and access to surgical care. Optimizing medical management is equally essential; strategies such as blood pressure control, anticoagulation reversal, and supportive care are vital for improving outcomes and minimizing complications. Post-acute rehabilitation and long-term recovery play key roles in shaping overall prognosis, emphasizing the need for a multidisciplinary approach involving neurology, neurosurgery, rehabilitation, and critical care teams to tailor treatment plans that enhance survival and quality of life. Early initiation of rehabilitation, encompassing physical, occupational, and cognitive therapy, should be prioritized to support neurological recovery and promote functional independence. However, uncertainties regarding the timing of interventions, selection criteria, and the comparative effectiveness of surgical versus medical management therapies still exist. Future research should identify biomarkers that predict hematoma expansion, treatment response, and long-term cognitive outcomes while rigorously assessing new therapeutic strategies such as minimally invasive techniques, neuroprotective agents, and targeted anti-inflammatory treatments. Ultimately, a balanced, patient-centered approach that integrates precise surgical techniques, optimized medical therapies, and comprehensive rehabilitation offers the best chance to improve both immediate survival and long-term recovery in patients with hemorrhagic strokes.
